# Practical Guidance for the Development of *Rosie*, a Health Education Question-and-Answer Chatbot for New Mothers

**DOI:** 10.1097/PHH.0000000000001781

**Published:** 2023-07-21

**Authors:** Heran Y. Mane, Amara Channell Doig, Francia Ximena Marin Gutierrez, Michelle Jasczynski, Xiaohe Yue, Neha Pundlik Srikanth, Sourabh Mane, Abby Sun, Rachel Ann Moats, Pragat Patel, Xin He, Jordan Lee Boyd-Graber, Elizabeth M. Aparicio, Quynh C. Nguyen

**Affiliations:** Department of Epidemiology and Biostatistics (Mss H. Y. Mane, Yue, and Moats Drs He and Nguyen), Department of Behavioral and Community Health (Drs Doig, Jasczynski, and Aparicio and Ms Gutierrez), and Public Health Science Program (Ms Sun), University of Maryland School of Public Health, College Park, Maryland; Department of Computer Science, UMIACS (Ms Srikanth and Dr Boyd-Graber), College of Information Studies (Mr S. Mane), and Department of Biology (Mr Patel), University of Maryland, College Park, Maryland.

**Keywords:** chatbot, health information, maternal and child health

## Abstract

Communities of color experience higher maternal and infant mortality, as well as a host of other adverse outcomes, during pregnancy and postpartum. To address this, our team is developing a free, user-friendly, question-answering chatbot called *Rosie*. Chatbots have gained significant popularity due to their scalability and success in individualizing resources. In recent years, scientific communities and researchers have started recognizing this technology's potential to inform communities, promote health outcomes, and address health disparities. The development of *Rosie* is an interdisciplinary project, with teams focused on the technical build of the application (app), the development of machine learning models, and community outreach, making *Rosie* a chatbot built with the input from the communities it aims to serve. From June to October 2022, more than 20 demonstration sessions were conducted in Washington, District of Columbia, Maryland, and Virginia, where a total of 109 pregnant women and new mothers of color could interact with *Rosie*. Results from the live demonstrations showed that 75% of mothers searched for maternity and baby-related information at least once a week and more than 90% of participants expressed the likelihood to use the app. Most of the participants inquired about their baby's development, nutrition for babies, and identifying and addressing the causes of certain symptoms and conditions, accounting for about 80% of the total questions asked. Mother-related questions in the community demonstrations were mainly about pregnancy. The high level of interest in the chatbot is a clear indication of the need for more resources. *Rosie* aims to help close the racial gap in maternal and infant health disparities by providing new mothers with easy access to reliable health information.

In the United States, there have been persistent disparities in maternal and child health and survival by race and ethnicity that are present even when socioeconomic status and education are controlled for.^1^ This is particularly alarming because it is estimated that 80% of maternal deaths are preventable in the United States.^2^ These racial disparities in maternal and child health outcomes are due to the interacting effects of systemic racism that hinder protective social determinants of health and barriers to affordable, high-quality, and culturally inclusive prenatal and postnatal health care.^3^,^4^ Reversing these negative health outcomes will require intervention at all levels of the socioecological model, including individual-level interventions that can provide low-barrier paths to medically accurate and culturally inclusive pregnancy and parenting information. Because of explicit and implicit bias across the medical field, women of color are also navigating relationships with health care providers that can place blame on mothers for negative health outcomes, which erodes the patient-provider relationship.^3^–^6^

Lack of straightforward, medically accurate information during pregnancy and postpartum can be particularly challenging for women of color whose concerns are frequently dismissed by providers, making it difficult to self-advocate as a patient.^7^ In addition, women can feel they do not have adequate time with or access to providers.^8^–^10^ They also worry about bothering the providers with or being judged for their questions.^7^,^11^,^12^ As a result, many turn to the Internet for knowledge and reassurance during pregnancy and their child's infancy.^8^,^13^,^14^ Frequently searched topics include breastfeeding, tongue-lip ties, infant health issues, and things they are uncomfortable talking to the provider about, such as postpartum depression and sex.^8^,^15^ However, the inconsistent quality or dubious sourcing of online information about pregnancy or infant care can lead to fear, particularly for first-time mothers.^8^,^13^,^16^

## Chatbots as a Promising Intervention

A chatbot is a computer program that can simulate the conversation capabilities of humans.^8^ With advantages such as instant responses and assistance with tasks, chatbots are commonly used as a customer service tool.^8^ Recently, there has been an increase in the need for technology, specifically chatbots, to answer health-related questions. The COVID-19 pandemic accelerated the development of chatbots for health assistance as the pandemic strained the health care system, with fewer health care providers being available to attend to patients' individual needs.^8^,^17^ A mixed-methods review conducted by Chua and colleagues^8^ to evaluate chatbots for pregnant and new parents indicated that, across 15 studies, these interventions were highly acceptable to participants, that chatbots are able to provide emotional and psychological support that can lead to reduced anxiety, and that chatbots would be improved using community-based reviews to increase cultural competence and the development of answers with a higher degree of specificity.

Furthermore, chatbots can help pregnant women with concerns related to stress, sleep, and breastfeeding.^8^ Chatbots can positively affect childhood development by providing parents with detailed, continuous information on infant stages of development, vaccination information, and healthy dietary practices.^8^

Recognizing the disparities in health care faced by women of color and the potential benefits of technologies such as chatbots, we worked over 3 years to engage community members and community partners in listening sessions about their interests and needs with a pregnancy and postpartum digital tool. On the basis of community feedback, we created and refined *Rosie*, a free question-answering (QA) chatbot available for pregnant and new mothers of color. *Rosie* provides mothers with resources and health information to support their and their baby's health during pregnancy and the first year of life. This article aims to describe the application's (app's) first phase of community-based evaluation after initial development.

## Methods

### Building *Rosie*'s knowledge base

A list of verifiable Web sources of information on maternal health and infant care was used to create *Rosie's* knowledge base. Sources included government agencies, professional medical organizations, and children's hospitals (N = 60). A corpus of documents about maternal and infant health was built by scraping text from these vetted Web domains using Trafilatura, a Web document processing tool that extracts text from HTML source code.^18^ Each Web document was then parsed into approximately 73 000 passages by splitting the text according to a collection of heuristics that retain sentence context.

These passages were edited as necessary to serve as answers to mothers' questions and were used in a question generation model, Probably Asked Questions (PAQ), to produce likely questions from users. The generated questions and their source passages were reviewed by annotators, who either edited both the question and the passage as necessary or discarded pairs that were unhelpful, inaccurate, or incomprehensible. The answers augmented the existing knowledge base by using multiple sources.

### Engineering components of *Rosie*

The underlying QA system uses a pretrained, unsupervised, dense passage retrieval (DPR) model, Contriever, designed to find the document that best answers a mother's question.^19^ The trained model first indexes documents by precomputing and storing their vector representations. When a mother asks a question, the model works by comparing the important features (vector representations) of the questions asked to the features of the documents and returns the passage that is the most similar. This process allows the model to return the most relevant and accurate answer to the mother's questions. Along with the answer passages, mothers are provided a link to the Web sources from which the passages were extracted to further contextualize our system's answer.

The Contriever model was evaluated by comparing the model's effectiveness with analogous models. Contriever learns to retrieve relevant passages through training with contrastive examples of passages from the same document and different documents, which means that the model can more effectively generalize its ability to retrieve text from unseen domains. In other words, Contriever has learned to recognize relevant text passages from irrelevant ones and can do so irrespective of the text's source or context.

**FIGURE 1:**
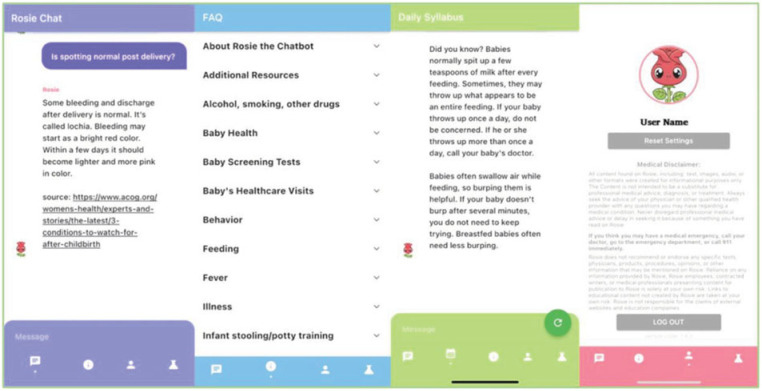
Retriever Model Performance Annotation Results This figure is available in color online (www.JPHMP.com).

In comparison, DPR, another retrieval model, is trained on positive and negative examples of relevance from select QA data sets based on Wikipedia.^20^ A DPR model was included in evaluation in 2 settings: *zero-shot*, in which the model was not further trained; and *fine-tuned*, in which the model was trained on the human-annotated examples of passage relevance. Annotators were presented with 127 questions sourced from *Rosie* community demonstrations and were asked to compare response passages from the models. Annotators selected response passages from the Contriever model 66% of the time, compared with the response passages from the fine-tuned and zero-shot DPR models. Therefore, the Contriever model was used in the implementation of *Rosie* (Figure 1).

The efficacy of a chatbot is also measured by its ability to identify the intention of the user, which was facilitated in our development of *Rosie* by applying an intent classification model. The classes of mothers' intentions included “greeting,” “goodbye,” “thank you,” and “ask *Rosie* a question” (main intent). “Physical health” and “mental health” intent categories were created to alert the team of potential mental health emergencies, such as severe anxiety, depression, and suicidal ideation, as well as physical health emergencies, such as severe bleeding and breathing problems. Upon detection of an emergency intent, besides notifying the team, *Rosie* simultaneously returns a message to the user with available emergency resources, for example, the National Maternal Mental Health Hotline.^21^,^22^ The intent classification architecture uses Rasa's Natural Language Understanding (NLU) and machine learning models that are trained on example texts and their assigned respective intents.^23^ The model assigns a probability to each predefined intent category and selects the intent with the highest score. The Rasa intent classification model was tested on a set of 547 real-world questions, including 389 questions from mothers at community demonstrations and 158 questions from the *Rosie* research team. The results indicated the model achieved a high accuracy of 98.5%.

Firebase Database, a Google cloud-based system, was used to store and monitor mothers' interactions with *Rosie*.^24^ Firebase was selected for its seamless integration with Flutter, a user interface (UI) software-building framework, as well as its reliable security features. The data stored included encrypted user ID, mothers' questions, chatbot responses, time stamps, and mothers' feedback. These data are used to further train the question-and-answer model and continually improve *Rosie*. Using an encrypted user ID and reliable and secure data storage comprises important steps to ensuring data confidentiality and privacy. In addition, a pulse check system was put in place that proactively detects server-related issues and app interruptions as they occur and notifies the research team through Slack, a cloud-based communication platform.

### Mobile app features

**FIGURE 2:**
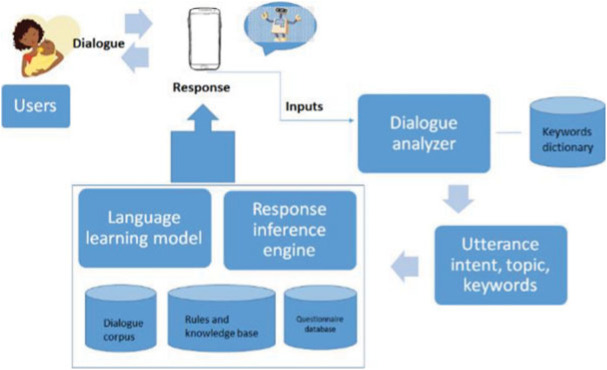
*Rosie* Chatbot Features (Left to Right: Chat Screen, FAQ Page, Daily Syllabus Notification, Disclaimer Page) This figure is available in color online (www.JPHMP.com).

A mobile app was identified as the most convenient option for mothers during community listening sessions because of ease of access and additional features such as push notifications for clinical visit reminders and daily health tips. Flutter is an open-source UI software platform built by Google and was integral for creating a multiplatform app compatible with both iOS and Android devices.^25^ Figure 2 displays some of the client-facing features of the app.

Logging into the *Rosie* app is done through Google authentication to enhance security by eliminating the need for users to create and manage passwords. A chat window page allows users to ask *Rosie* any questions related to pregnancy, postpartum health, infant health, and infant care. In addition, a rating system was implemented with clickable thumbs-up or thumbs-down buttons to garner mothers' feedback for each of *Rosie*'s responses.

At first use, mothers are prompted to enter an approximate (not exact) due date or baby's birth date to ensure participants' confidentiality. The dates provided were then used to generate a daily health tip based on pregnancy week (for pregnant mothers) or the infant's age (for mothers who had already given birth). Daily facts and health tips include information about different stages of development, nutrition, physical activity, safety, and developmentally appropriate ways to interact with mothers' infants. Mothers receive daily tips via push notifications, and health tips from the past 7 days are saved and displayed on an app page called “Daily Syllabus” for mothers' reference. Furthermore, a Frequently Asked Questions (FAQ) page was created with question-answer pairs organized by clickable tabs, focusing on the most popular topics including postpartum symptoms and complications, infant feeding, and developmental milestones. For each FAQ, a clickable source Web link was provided.

### Connecting it all: *Rosie*'s overall architecture

**FIGURE 3:**
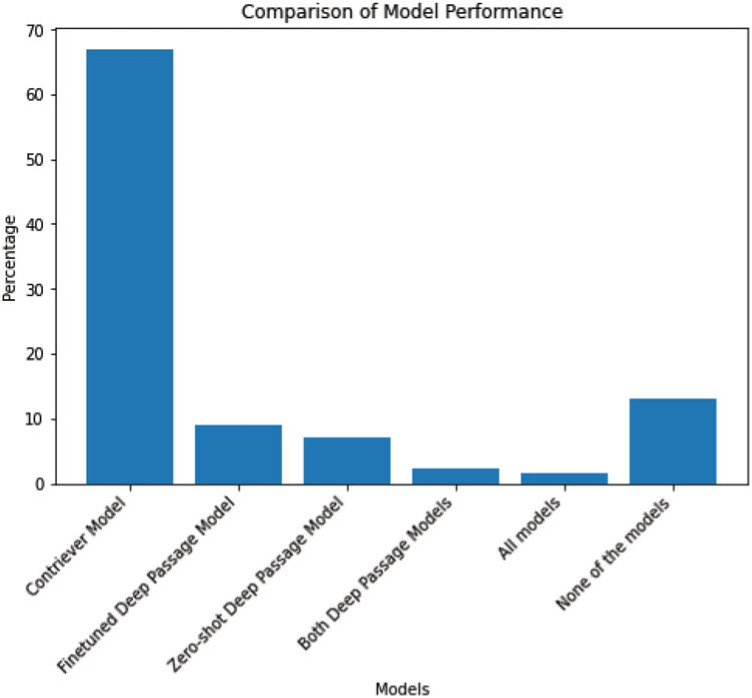
*Rosie* End-to-End Model Architecture This figure is available in color online (www.JPHMP.com).

An end-to-end system architecture (Figure 3), which consists of a user-facing mobile app (“client”), a backend server, and a QA system, was created. After a mother (user) inputs a question, the backend server receives and processes it before directing it to the QA system where natural language processing systems understand the query and retrieve the relevant information from the knowledge base and return a response via the client to the mother.

### Community-engaged testing of the *Rosie* app

The development of *Rosie* has always centered on the lived experiences and needs of mothers of color. Following a series of initial listening sessions and consultations with mothers of color, our team conducted community demonstrations to understand the sources mothers use to obtain health information, the topics they were interested in learning more about, and mobile app functionalities they would like. As a result, we were able to expand the corpora of information relevant to the intended audience and improved the features *Rosie* offers. Our community-engaged strategy also allowed for testing and developing the iterations of the chatbot. The research team reached out to community-based organizations and events, such as farmers' markets, citywide festivals, and organizations providing financial or material assistance to parents, to gain iterative feedback on *Rosie*'s development. From June to October 2022, we completed more than 20 demonstration sessions where pregnant women and new mothers of color could interact with the *Rosie* chatbot app. Eligible community members were female, 14 years and older, currently pregnant or parenting a child younger than 3 years, and part of a racial or ethnic minoritized group (African American or Black, Asian, Native American or Alaska Native, Native Hawaiian or other Pacific Islander, and/or Latinx).

On-site demonstrations were led by 2 to 3 research team members who would approach pregnant or new mothers to introduce the app. Mothers were provided with an iPad with *Rosie* downloaded where they could ask maternal and child health-related questions. After interacting with *Rosie*, mothers were then invited to complete an optional postdemonstration survey loaded on the same iPad to gain feedback on usability, feasibility, and acceptability of the app. The research team member read the questions out loud to participants and recorded participants' answers verbatim in the iPad. A total of 109 mothers completed the survey following demoing *Rosie*. To accommodate Spanish-speaking participants, bilingual team members were also present at the demonstrations to assist with survey demonstration and translation. Our decision to select these community sites was motivated by our desire to engage with a diverse range of women of color and capture their experiences, feedback, and questions while interacting with *Rosie*.

## Results

Among the demonstration participants (n = 109), 66% were iPhone users and 32% were Android users. About 75% of mothers said they searched online for maternal and infant health information at least weekly. More than 96% of participants expressed they were likely or very likely to use *Rosie*. Table 1 displays responses to survey questions. When given the opportunity to ask *Rosie* questions, most participants asked questions about their baby's health and care (generally baby's development and nutrition), accounting for about 80% of the total questions asked. Maternal health–related questions were mainly about pregnancy, such as labor, abortion, and mental health. Example questions are presented in Table 2.

**TABLE 1:** Demonstration Feedback Survey Questions and Response

Which mobile device do you use?	
Android	33.3%
iPhone	65.8%
Other	0.8%
How often do you typically search the Internet for maternity and/or baby-related information?	
Multiple times a day	31.13%
Once daily	10.38%
Weekly	33.96%
Monthly or less	24.53%
How likely are you to use *Rosie*?	
Very likely	61.76%
Likely	34.31%
Not likely	3.92%
How often would you use *Rosie*?	
Multiple times a day	24.27%
Once daily	13.59%
Weekly	45.63%
Monthly or less	16.50%

**TABLE 2:** Example of Question From *Rosie* Demonstration Participants

Question Categories (Question Counts)	Question Examples
Development (81)	What age do babies stop growing? How to introduce solid foods at 6 mo? How soon should I expect my baby to begin teething?
Symptoms/conditions (33)	Can a baby with an inverted heart be nebulized? Why is my newborn vomiting after breastfeeding? Why does my back hurt?
Safety (13)	At what age can I turn my convertible car seat forward? Why should I put my baby on its back to sleep? What to do if my child gets bitten by a bug?
Parenting (10)	What can I do so my child behaves properly? What should I do if my baby cries a lot? How often should I change the baby's diapers?
Pregnancy (14)	How do I know if I am pregnant? How can I take care of myself when pregnant? Is DC able to do abortions?
Medical (6)	How many milligrams of ibuprofen can I give my baby per kilogram of weight? How much vitamin D supplement do babies need a day?
General (5)	What clothes should I buy for my 1-y-old? What is the probability that a pregnancy test is positive 2 wk after a miscarriage?
Mental health (4)	Do I have anxiety? Why do I feel mad after giving birth?
General parenting (2)	How often do I need to bathe my baby? What is it like after having a baby for the first time?
Postpartum (1)	Is it normal for your period to be irregular postpartum?

Feedback received during the demonstrations of the app has been encouraging, with many women expressing excitement to be a part of the project and the need for similar resources. Participants noted feeling secure with using familiar brands such as Google and Gmail being used as their login information. In addition, participating community-based pregnancy and motherhood support organizations expressed that apps such as *Rosie* address a critical problem contributing to maternal and infant health disparities by providing immediate access to accurate information during situations when other support systems are unavailable.

A community-engaged approach was essential to informing a number of improvements to *Rosie*, including addition of more topics to the *Rosie* knowledge bank; enhanced multilingual capacity of *Rosie* to deliver responses in Spanish; daily health tips that users receive through app push notifications; and a pulse check that would notify the research team if the *Rosie* app went down.

## Discussion

Our interactions with community members and previous research underscore a significant need for improved access to information and resources related to maternal and infant health within communities of color.^13^–^16^ Demonstration participants were curious as to how artificial intelligence (AI) in particular works, and participants were given an overview of how their positive and negative responses to generated answers helps refine the knowledge base and models used by the development team. Our community-driven approach helps ensure that *Rosie* is tailored to the needs of the community.^8^

Likewise, chatbots facilitate a more interactive and iterative development of content and app features in a way that is more feasible than other health communication options, such as a Web site without interactive features or pamphlets. From this phase of our project, we have found users also appreciate the iterative development process and that their feedback is integrated into the app's answers. Also, it is an acceptable intervention to refine and test further through our planned pilot study and a randomized controlled trial. Similarly, our experience demonstrating the *Rosie* app can serve as a guide to other developers on how to manage an iterative development process and maintain quality and scalability of the app. Chatbots can also protect users' privacy and provide a degree of confidentiality, which is important for reproductive health care and was a feature demonstration participants were curious and concerned about.^8^,^17^

Chatbots are not without limitations. Although chatbots imitate human conversations, they are merely AI systems that can only go as far as the information they were trained on. They are not able to personalize their interaction with their users the way that medical professionals can. In addition, given entrenched societal biases and systemic racism, which extend into the medical and science communities, chatbots run the risk of perpetuating those biases by building them into their knowledge bank. The *Rosie* research team devoted considerable effort to vetting government and accredited medical sources for health information. As an added safeguard, the research team included a clinical advisory board that provided guidance on every phase of *Rosie*'s development. The board reviewed sources, provided guidance on the structure and content of the knowledge base and curriculum, helped decide on participant incentives, and shared their experiences meeting the medical needs of women of color. Finally, chatbots may not be accessible to everyone. *Rosie* may not be accessible for mothers who may not have access to smartphones and tablets, have limited access to Wi-Fi or the Internet, or mothers who may have technological literacy limitations.

To help mitigate these barriers, developers should be mindful about developing user-friendly apps and creating an app that can be easily navigated by its users. This includes being cognizant about the app's features and navigation, the language used, as well as drafting clear and understandable instructions for how to effectively use and get the most out of the app. Developers could also consider making the app available across multiple devices including computers. Alternatively, integrating the app into services such as Short Messaging Services (SMS), a text messaging service that does not require an Internet access data plan or Wi-Fi, could be useful for reaching more disadvantaged people. Furthermore, *Rosie* occupies a relatively small amount of disk space of 58.1 MB and the monthly data usage is about 20 MB. Developers should be mindful about creating apps that are more storage and data efficient.

The community demonstrations of *Rosie* highlighted a need for health information as mothers routinely searched online for health information but noted their needs for culturally competent vetted sources. In addition, mothers were very receptive to using *Rosie* and most would plan to use *Rosie* at least weekly. A randomized controlled trial is planned to test the impact of *Rosie* on reducing postpartum depression, reducing emergency department visits for infants, and increasing attendance at well-baby visits.

**Implications for Policy & Practice**

- The integration of health care with technologies such as *Rosie* has the potential for improving access to health information, particularly for underserved and marginalized populations.
- Health chatbots can be tailored to address the specific health needs and cultural contexts of various communities.
- This article aims to offer high-level project planning guidance on how to approach community-engaged development of a QA chatbot. It is intended to inform various considerations of the broad overview of key principles and strategies for designing user-centered apps.
- Chatbots can provide policy makers, health care administrators, and providers with data on the needs of underserved and marginalized communities. It is also important to evaluate the use of data acquired through chatbots to reinforce existing and establish new policies that will continue to protect the privacy and security of the users.
